# Supplementary figures and images for: Comparison of functional-oil blend and anticoccidial antibiotics effects on performance and microbiota of broiler chickens challenged by coccidiosis
Source: PLoS One. 2022 Jul 6;17(7):e0270350. doi: 10.1371/journal.pone.0270350 (PMC9258845; doi:10.1371/journal.pone.0270350)

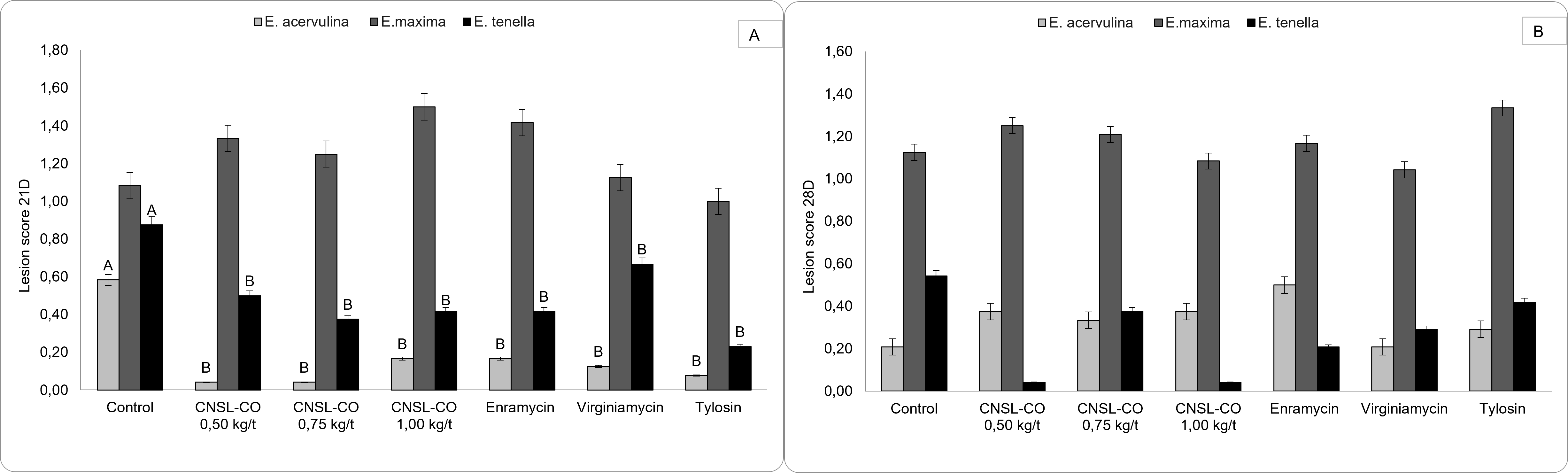

Supplement: S1 Fig — Lesion score in coccidiosis challenged broilers at 21 (A) and 28 (B) days of age—7 and 14 d after challenge, respectively. CNSL–Castor (US Patent N. 8377,485; Oligo Basics Ind. Ltda., Cascavel, Parana, Brazil). Means with different letters differ statically as per the Student–Newman–Keuls method. (PNG) [file pone.0270350.s001.png]

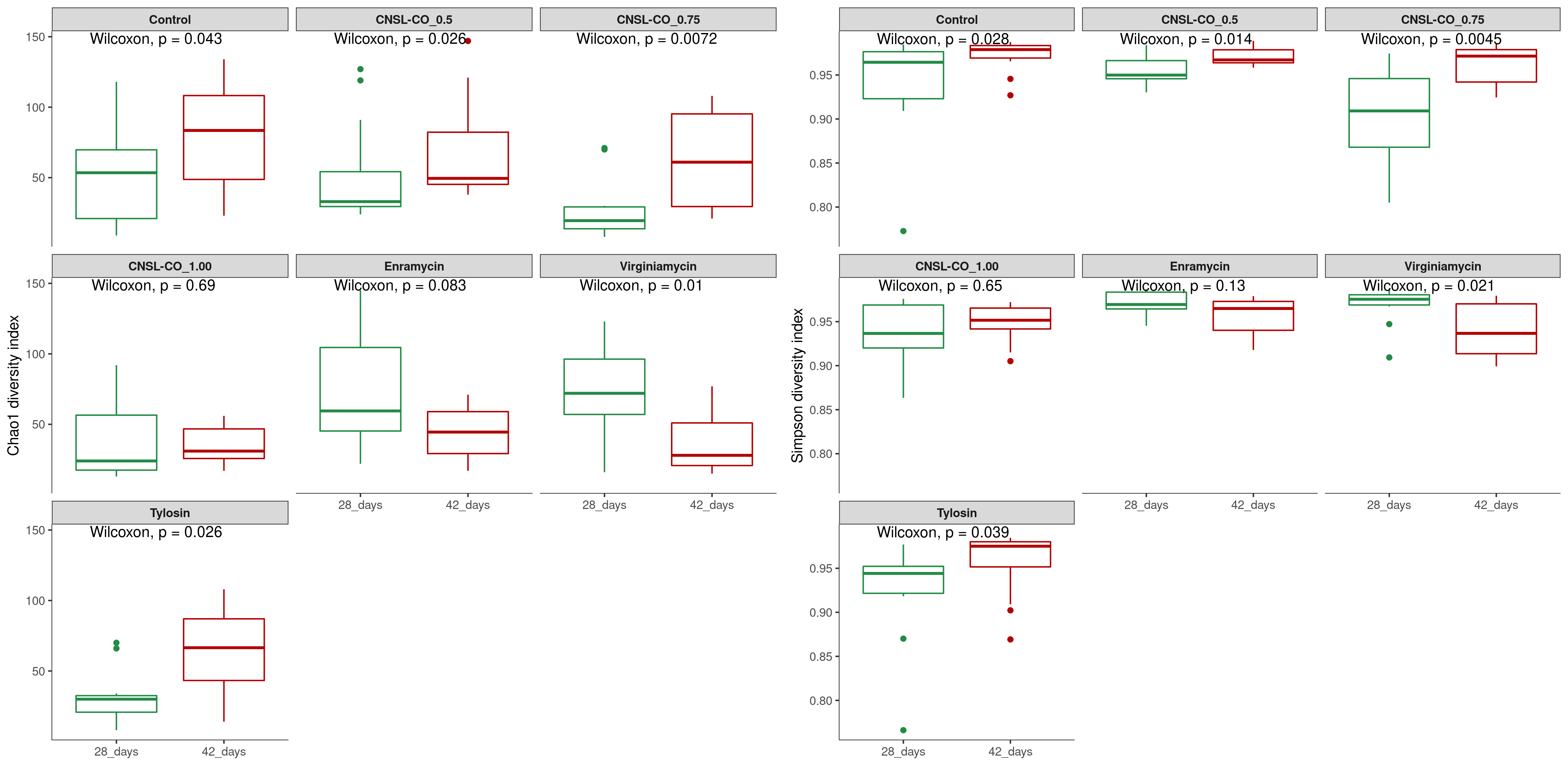

Supplement: S2 Fig — A, B. Comparison of alpha diversity for each additive between time points using CHAO -1 (A) and Simpson (B) index of broilers cecum samples at 28 and 42 d of trial. The study tested seven feed additives: enramycin (8 ppm), virginiamycin (16.5 ppm), tylosin (55 ppm), CNSL–castor oil (CNSL-CO) in different doses (0.5, 0.75, and 1.00 kg/t), and the control diet (without additives). (PNG) [file pone.0270350.s002.png]

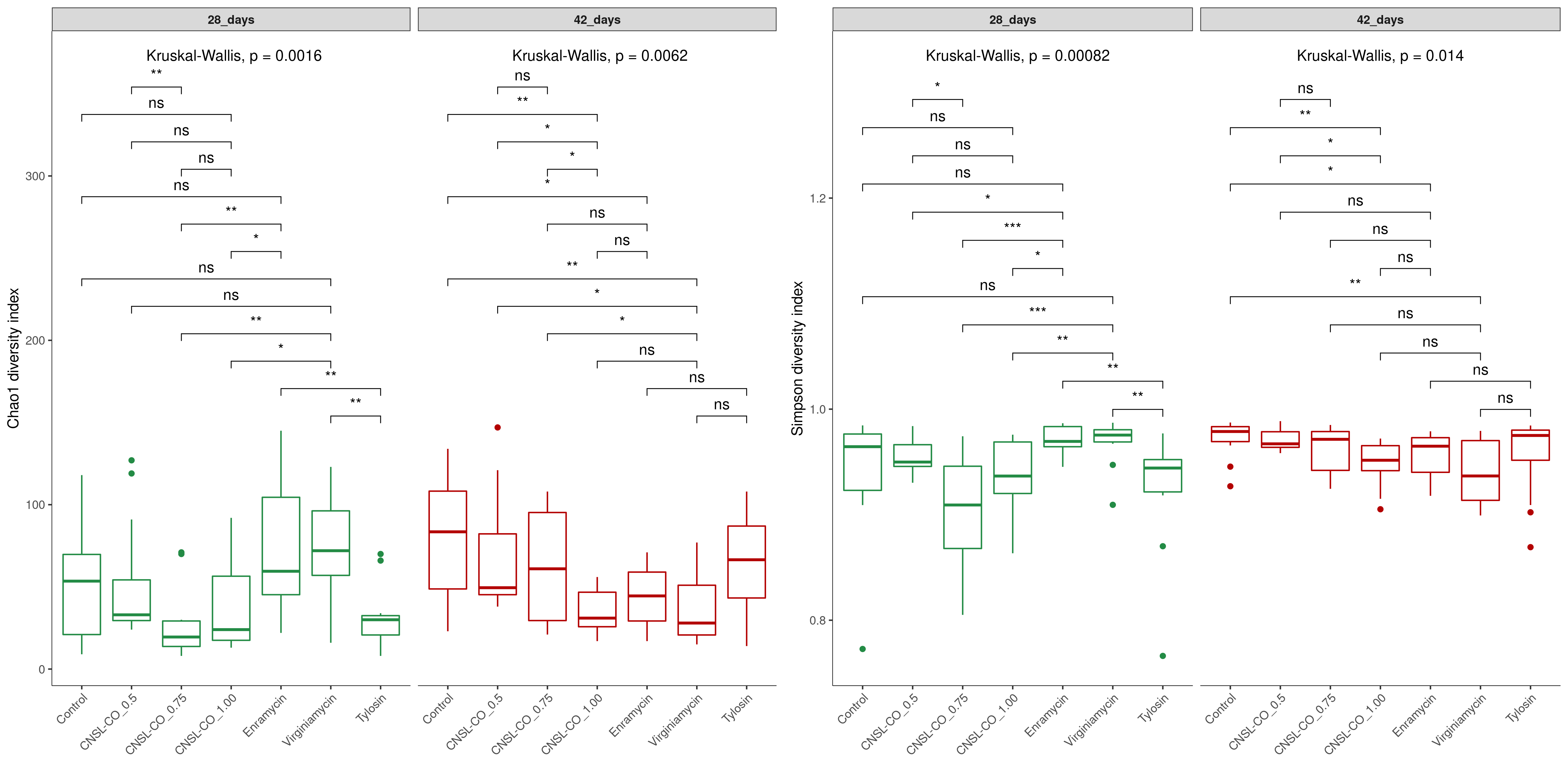

Supplement: S3 Fig — A, B. Comparison of alpha diversity at each time point using CHAO -1 (A) and Simpson (B) index of broilers cecum samples at 28 and 42 d of trial The study tested seven feed additives: enramycin (8 ppm), virginiamycin (16.5 ppm), tylosin (55 ppm), CNSL–castor oil (CNSL-CO) at different doses (0.5, 0.75, and 1.00 kg/t), and the control diet (without additives). (PNG) [file pone.0270350.s003.png]
