# Supplementary material for: Comparison of functional-oil blend and anticoccidial antibiotics effects on performance and microbiota of broiler chickens challenged by coccidiosis
Source: PLoS One. 2022 Jul 6;17(7):e0270350. doi: 10.1371/journal.pone.0270350 (PMC9258845; doi:10.1371/journal.pone.0270350)
Supplement: S1 Table — (DOCX) [file pone.0270350.s004.docx]

Suplementar table 1. Ingredient formulas and chemical composition of experimental diets according to the rearing period.

| Ingredients, % | Starter  (1-28 d) | Grower  (29-35 d) | Finisher  (36-42d) |
| --- | --- | --- | --- |
| Corn | 557.60 | 602.80 | 603.80 |
| Soybean meal 45,5% | 380.08 | 328.00 | 328.00 |
| Soy oil | 26.00 | 33.80 | 33.80 |
| Dicalcium phosphate | 17.60 | 11.50 | 11.50 |
| Limestone 37% | 6.90 | 11.33 | 11.33 |
| Salt | 5.10 | 4.70 | 4.70 |
| DL- Methionine 99% | 2.70 | 2.30 | 2.30 |
| L - Lysine HCL 78% | 0.70 | 1.10 | 1.10 |
| Choline Chloride 60% | 0.80 | 0.80 | 0.80 |
| Vitamin - Mineral Premix¹ | 0.70 | 0.70 | 0.70 |
| Semduramycin + Nicarbazine | 0.50 | 0.50 | - |
| Monensin sodium | 0.25 | 0.25 | - |
| Inert/Antibiotics/CNSL-Castor oil^2^ | 1.00 | 1.00 | 1.00 |
| Total | 1000 | 1000 | 1000 |
| Calculated composition | | | |
| Metabolizable energy (Kcal/kg) | 3000 | 3100 | 3100 |
| Crude Protein (g/kg) | 219.30 | 198.50 | 198.50 |
| Dig. lysine (g/kg) | 12.60 | 10.50 | 10.50 |
| Dig. methionine (g/kg) | 5.90 | 5.00 | 5.00 |
| Available P (g/kg) | 4.4 | 3.20 | 3.20 |
| Calcium (g/kg) | 8.70 | 9.4 | 9.4 |
| Sodium (g/kg) | 2.20 | 0.21 | 0.21 |

¹ Mineral and vitamin contents in experimental feeds, mg/kg feed: Vitamin A 10,000 UI, Vitamin D3 2.300 UI, Vitamin E 33.5 UI, Vitamin K3 1.8 mg, Thiamine 2.4mg, Riboflavin 6.0 mg, Pyridoxyne 3.2 mg, Cobalamin 0.032 μg, Pantothenic acid 18.8mg, Biotin 0.09mg , Niacin36,1mg, Folic acid 0.85mg, Selenium 0.39, Iron 60.0 mg, Zinc 78.7 mg, Manganese 85.0 mg, Copper 12.0 mg, Iodine 1.2 mg

^2^ Each treatment used an antibiotic: enramycin (8 ppm, MSD Animal Health), virginiamycin (16.5 ppm, Phibro Animal Health, Teaneck, NJ, USA) and tylosin (55ppm, Elanco Animal Health, Greenfield, IN, US); or different doses of CNSL - Castor oil (0.5 kg/t; 0.75 kg/t; 1.00 kg/t). All antimicrobial doses are recommended at subtherapeutic levels for disease prevention or growth promotion by respective manufacturers.
