# Supplementary material for: Comparison of functional-oil blend and anticoccidial antibiotics effects on performance and microbiota of broiler chickens challenged by coccidiosis
Source: PLoS One. 2022 Jul 6;17(7):e0270350. doi: 10.1371/journal.pone.0270350 (PMC9258845; doi:10.1371/journal.pone.0270350)
Supplement: Attachment — Submitted filename: PONE - D 22-04-04312 OBSERVATIONS.docx [file pone.0270350.s007.docx]

PONE-D-22-04312

TITLE: Comparison of the use of a blend of functional oils with antibiotics associated with anticoccidials on the performance and microbiota of broiler chickens challenged by coccidiosis

I´d like to understand where authors saw an associations of antibiotics and anticoccidials, because I can´t see it. The antibiotics were used in almost all the period alone. One in one week monensin was used together with the antibiotics . So, what do you mean with this “association” ? For e associated means together, with, and not after.

Abstract e M&M it was not 2520 divided in 12 pens, but in 84 pens

Line 33 28 D). The challenge was carried out by inoculating broiler chickens with

34 sporulated *Eimeria tenella*, *Eimeria acervulina,* and *Eimeria maxima* oocysts via35 oral gavage. In addition to performance parameters, intestinal contents were36 collected at 28 and 42 days of age for microbiota analysis by sequencing 16s37 rRNA in regions V3 and V4 using the Illumina MiSeq platform

It is lacking- When, in what age was done the inoculation?

Line 31-All treatments received semduramicin + nicarbazin (500g/t) and sodium monensin (100ppm) in the initial live phase (0 to

28 D). In the M&M you say that monensin was from 29 days until 35 (Line 122).

“There was increasing diversity from 28 D to 42 D using the three indices “– you did not told us what indices are you talking about…

“There was increasing diversity from 28 D to 42 D using the three indices, which were significant only for the Simpson index and showed higher time dominance at 42 days”- in what treatment? Only due to an age influence? In other words, why there was an increase in diversity?

Line 39 “In the evaluation at 21 days, the broilers that received tylosin had…! Why a 21 day response if you´ve changed diet only at 28 days? I onçy understood this response nowing challenge was at 14 days. So, rewrite saying that “ after one week of challenge or something like this…”

“at 42 days, it had the lowest”. It had the lowest what??

“Tylosin showed better performance indices of chickens challenged by coccidiosis.” You did not tell us if there was some treatment WITHOUT challenge. How can we understand?

” . On the other hand, the antibiotics also increased the abundance of bacteria producing short-chain fatty acids from 28 days” – what do you mean with from 28 days? AFTER 28 days? Or from 28 up to 42 days.

Introduction

Line 67 “significant increase in its proliferation in the small intestine and it can cause necrotic enteritis [2,3]

Line 74 - and direct inflammatory [5]. How AGP causes inflamation? It is the contrary.

Line 110 - the birds could have euthanized?

111 was no specific euthanasia situation : the coccidiosis infection responded to the…

M&M

Line 13. The experimental period lasted 42 days and broilers were Weekly weighed, measured feed intake was measured, and calculated feed

Conversion were calculated [11] Please change place (from Line 124 to here). The information is loose up there.

Line 188 The performance responses separated according to the periods 1 to 21 ( no :)

191 presented more significant weight gain ?

... presented greater weight gain , with a statistical significance (is that you mean???)

Line 102- while the other treatments demonstrated an intermediate

193 behavior, and consequently, had higher live weight and better FCR (p < 0.05). You wrote that the OTHER TREATMENTS had better FCR an I think you wanna mean that tylosin treatments had netter FCR. Rewrite the sentence, please.

194 The control group showed worse feed conversion (P < 0.05). Rewrite please. Be more direct.

Line 200 –“The following period was completed 14 days after the challenge by *Eimeria* spp (14 to 28 D)”… you´ve told about 3 periods (The performance responses separated according to the periods- 1 to 21 days, 21 to 28 days, 28 to 42 days, and a total period of 1 to 42 days.) . What following period? Not even in Table 1 is this period.

219 0.05). Consequently, the pattern for live weight at 42 days was identical to weight gain (identical are the same numbers)

Line 226 In the first week after infection, the broilers submitted to the control

227 treatment had a higher lesion score for *E. acervulina* and *E. tenella* when

228 compared to the other treatments (p < 0.05). Results not shown???

246 The same trend??? occurred for all observed indices (Fig. S2 A and SB)

260 Comparing the different additives at each time point, we observed

261 statistically significant differences between them (Kruskal-Wallis, P < 0.05). Each262 treatment was pairwise compared using the Wilcoxon test corrected by the FDR263 (False Discovery Rate). This is not results. It is M&M

At 28 days, there were no significant differences compared to the control (Fig. 3). At 42 days, the control differed from treatments with virginiamycin, CNSL–castor oil 1.00kg/t, and enramycin (Fig. S3A e S3B). I did not find Fig S3A and Fig S3B.

Why the comparison towards control treatment? In my opinion, or you explain all differences ( a lot, may be) or just say about the existence of differences,

277 samples were scattered with overlapping ellipses (Fig. 4A). At time 42,?

At 42 days of age?

280 treatments at each studied time,

281 are not similar. Comparing each treatment at different times (periods?// days??) times is not an appropriate term

283 time 28 days to time 42 days (Adonis, p < 0.05) (Fig. 4B and Table 2). Time again

328 and 1kg/t did not differ from the treatment with enramycin and virginiamycin, with a better feed conversion when compared to the control treatment. Here you are talking about the comparison between enramycin , virginiamycin x castor oil. Leave control out this comparison.

Line 348- On the other hand, ionophores are considered antibiotics of non-medical importance for human health [21] (.periodo) In animal production,end point…

353 used antibiotics had similar zootechnical performances… It is not an usual word in English

Line 354 - …However, cases of parasite resistance have already been

355 reported due to the use of these substances for an extended period [22]. What substances? Antibiotics or ionophores?? Not clear. Use the expressions “the last, the former”

366 This study observed that even using anticoccidials, there was a higher

367 lesion score for *E. acervulina* and *E. tenella* for the control compared to the other368 treatments, which demonstrates beneficial action of CNSL-Castor oil during369 coccidiosis challenge, similar to the effect of antibiotics.

Wrong sentence. What do you wanna say? If anticocidials were totatlly efficient we should expect NO lesion score. So, even using anticoccidial it did not work perfectly.

And after you continuous… However, alternatives to

370 antibiotics can become ineffective in an environment with fewer biosafety. However? But it did not work perfectly. Why however?

373 In this study, added the use of anticoccidials in the initial phase FOLLOWED BY CNSL-Castor oil allowed a similar performance to antibiotics.

The text has to be clear that was a sequence not an addition, not an association.

377- This study used lower levels of CNSL-Castor oil (0.75 and 1.0 kg/t)

376 compared to previous studies, evaluating its isolated effect, demonstrated its beneficial effect with 1.5 kg/t [8,29].

English is not correct. Rewrite the sentence

377 found no synergistic effect between oregano essential oil and monensin at low doses. The combination of the two additives showed similar effects to the substances used separately in broilers challenged by coccidiosis.

Why do youpoint this? In your artile there was no associations to see synergistic effect. This citation is lost in the contend.

377 found no synergistic effect between oregano essential oil and monensin at low doses. The combination of the two additives showed similar effects to the substances used separately in broilers challenged by coccidiosis.

This part of the article is discussion, not introduction.

386 The microbial richness and diversity of the gut are closely related to broiler ´s health [34].

391- In this study, when evaluating at 28 and 42 days of age, it was

observed that even within treatments containing antibiotics, there was a change in microbial diversity, except for enramycin and CNSL-castor oil 1kg/t. How is this change? Incresed?? Decreased?

Line 394- 1.5kh/t of CNSL What unit is that?

411- However, it has been able to maintain intestinal homeostasis and negatively correlated with Proteobacteria, which are mostly facultative or obligate anaerobes with flagella or with the ability to move by gliding, including pathogenic species such as Salmonella and Escherichia. In broiler chickens, it has been associated with poor performance [41].

Sentences difficult to understand. Rewrite, please. Readers cannot understand if Proteobacteria is good or not

439 showed greater abundance for treatments with Tylosin and Enramycin (when) compared to the control

444 butyrate-producing bacteria [47,48]. Robinson et al. [49]) used tylosin and

445 observed that Tylosin and Enramycin increased

Tylosin with capital letter or not?

449- The Erysipelotrichaceae family showed greater abundance 450 at (wrong preposition) the 0.75 and

454- Corroborating the results of this study, which found

455 that lower levels of CNSL-castor oil were associated with anticoccidials and

456 increasing abundance at 42 days in their absence.

The structure of the sentence is wrong.

Do you mean the study of Vieira et al corroborate the results of the present study? And what do you intend to say about … ” lower levels of CNSL-castor oil were associated with anticoccidials and increasing abundance at 42 days in their absence.”? How low levels of castor oil are associated with anb? They increase the abundance of what?

458- abundance increase? How you know it is an increase since you did not measure BEFORE 28 days?

468 The use of different subtherapeutic doses of growth-promoting antibiotics

469 has different effects on bacterial adhesion of the cecum , (coma) [51] especially when470 added to the use of anticoccidials.

472 microbiota compositions can result in similar growth performances, as seen in this research [2] . I don´t think this is a corret manner to cite the article

Conclusion On the other hand, the antibiotics increased

481 the abundance of bacteria producing short-chain fatty acids at 28 days. For me, this is not clear in you discussion and it shows up in the abstract an in the conclusion. Or you emphasize in the discussion and in the results or you take it out.
